# Landslide susceptibility assessment using the Weight of Evidence method: A case study in Xunyang area, China

**DOI:** 10.1371/journal.pone.0245668

**Published:** 2021-01-25

**Authors:** Yanbo Cao, Xinsheng Wei, Wen Fan, Yalin Nan, Wei Xiong, Shilin Zhang

**Affiliations:** 1 Department of Geological Engineering, Chang’an University, Xi’an, China; 2 Key Laboratory of Western China Mineral Resources and Geological Engineering, Xi’an, China; 3 China Electronic Research Institute of Engineering Investigations and Design, Xi’an, China; Universidade Federal de Uberlandia, BRAZIL

## Abstract

The aim of this study is to provide a landslide susceptibility map of the Xunyang District of a mountainous terrain, at the southern part of the Qin-Ba Mountain Region, which has been highly exposed to widely distributed shallow landslides over the past few decades. The Weight of Evidence (WoE) method was adopted in this research considering both the presence of a certain landslide causative factor class and the absence of remaining classes, which was used for determining a clearly spatial correlation between a landslide occurrence and the causative factors. Intrinsic factors, including geomorphological factors, geological factors, and river flow networks, and external factors of anthropogenic engineering activities in terms of density of road network were all considered and involved in the Geological Information System (GIS) environment for reconstructing the thematic layers of factor dataset. Significant assumptions prior to the analysis were emphasized to ensure conditional independence between each pair of factors for this bivariate statistical approach. In addition, a detailed landslide inventory map was constructed through field investigation and a remote sensing interpretation process at a scale of 1:50000. The thematic layers and landslide map were overlapped to obtain a spatial statistical relationship by using the frequency ratio method. At last, the validation process for the derived susceptibility map was conducted by applying the ROC curve, indicating that more than 90% of the landslides were in categories of high and moderate susceptibility zones. The causative factor classes, including the slope angles ranging from 20 to 40°, strong weathered and fractured strata, and road network density were identified to considerably influence the landslide distribution in the study area. The results have proven to be significantly meaningful for landslide hazard risk mitigation and land use management for the local authorities responsible for these fields.

## 1. Introduction

An increasing number of researches on analyzing rainfall-induced landslides have been investigated in recent years, attributable to the extensive distribution of this type of geological hazard in various geological settings worldwide [1, 2]. In the Qin-Ba Mountain Range of the southern part of Shaanxi Province, China, rainfall-induced landslides have occurred more frequently over the past few decades following the rainy season each year and have posed a considerable threat to the local infrastructure and traffic safety under rapid urbanization. Particularly, a total number of 556 recently occurred landslides were identified and investigated after an extreme rainfall event occurred on July 18th, 2010 in the Xunyang District, located in southern Qinling Range, and landslides resulted in a lot of casualties and economic losses [3]. Thus, a reliable and suitable landslide assessment method is urgently needed to predict the probability of landslide occurrence in the future and reduce the hazard risks in a specific region, together with the goal of providing evidence for strategic planning for the local department of land use.

Varnes (1984) [4] provided a definition of landslide hazard zonation, which can be delineated in terms of the probability of occurrence of the landslides in a given area and a given period. However, landslide susceptibility mapping has always been a great challenge because detailed knowledge on landslide inventory and different triggering process are scarce. Several techniques have been previously and successfully applied in the field of landslide susceptibility assessment, generally comprised of empirical methods, statistical methods, and deterministic methods [4–10]. Later, some new techniques, for example, the Artificial Neural Networks (ANN) and Machine learning were introduced for mapping landslide susceptibility zonation [10, 11]. For example, Yilmaz [11] conducted a landslide susceptibility analysis by comprehensively comparing three different quantitative methods, including frequency ratio, logistic regression and artificial neural networks. Particularly, the superiority and drawbacks of each method was presented and described. The selection of an appropriate method for the landslide evaluation relies on the aim and scope of the research. When dealing with a specific landslide initiation process, complicated constitutive equations for slope materials and detailed boundary conditions are imperative in corresponding mathematical calculation procedures in understanding and modelling the triggering of a landslide process. When mapping regional landslide probability by using deterministic methods, several simplification procedures, including assumptions regarding homogeneous slopes and infinite slope patterns should be considered, for analyzing the slope stability [12–14]. Although explicit landslide triggering process can be well understood by using the numerical modelling methods, however, it is often difficult to acquire numerous parameters of each soil property under both saturated and unsaturated conditions in a wide area, and cumbersome computational procedures for solving a series of numerical models are time-consuming. If the research goal was mainly focus on the identification of regions prone to landslides or performing quantitative assessment on how geological factors affect spatial distribution of landslides, regional landslide susceptibility assessment (RLSA) represents an ideal tool for evaluating the spatial probability of potentially damaging landslide occurrences by classifying sections with different landslides susceptibility or probability levels on a map in a given area. The characteristics of past landslides in the area can be adopted to determine the probability of the landslides considering similar geological effects. Therefore, the map of the landslide susceptibility helps to identifying the area in which the researchers put more attention in performing detailed investigation on landslides and for further regional landslide risk analysis. Regarding spatial landslides assessment, the knowledge-driven and data-driven methods have been fully developed in previous years, and current artificial intelligence methods have gained more attention than ever before. Compared with knowledge-driven methods which depend upon specific expertise, data-driven methods, specifically referring to statistical methods including bivariate and multivariate statistical methods, have been widely considered as efficient and appropriate techniques for quantitatively characterizing statistical relationship among a series of geological factors and the spatial distribution patterns of landslides at a regional scale [15, 16]. The influence of each landslide controlling factor can be quantitatively determined according to statistical dataset involving historical landslides occurrences and related geological factors in a given area. The weight of evidence (WoE) method has been successfully applied in hazard mapping due to its predictive power of each causative factor and considers the relationship among both the presence of each factor class and the absence of remaining sub-classes and the landslide events. WoE method employs the Bayesian probability model in a log-linear form, and each factor can be linearly superposed in the GIS environment. Other reasons for adopting the WoE method rely on the independent characteristics of each variable of landslide factor. Thus, the effect of each causative factor can be independently considered and a decreasing number of variables can be ascertained in a landslide susceptibility analysis, compared with other statistical methods, for avoiding possible existence of mutual correlated factors, otherwise, which might derive unreliable results. Therefore an optimal set of landslide causative factors (only a few numbers of factors) can be selected in this analysis by introducing an additional procedure of testing the conditional independence for each pair of factors, avoiding impacts from mutually correlated factors [17], and was thus adopted in this research.

The purpose of this research is to create a reliable landslide susceptibility map for the Xunyang District in the southern part of the Qinling Mountain Range, China, to reduce potential landslide hazards faced by local urbanization. In this region, results from field investigation have indicated that fractured/jointed phyllites of epimetamorphic strata and multi-tectonic fractures presented in the area largely control the slope stability, and, hence, the spatial distribution of landslide occurrences. To provide a better understanding of the interaction between them and attempt to predict the probability of potential landslide occurrences, the Weight of Evidence method (WoE) has been adopted for the quantitative zonation of the area with different probability levels of landslide occurrence.

## 2. Geological backgrounds and landslide inventory

**Fig 1:**
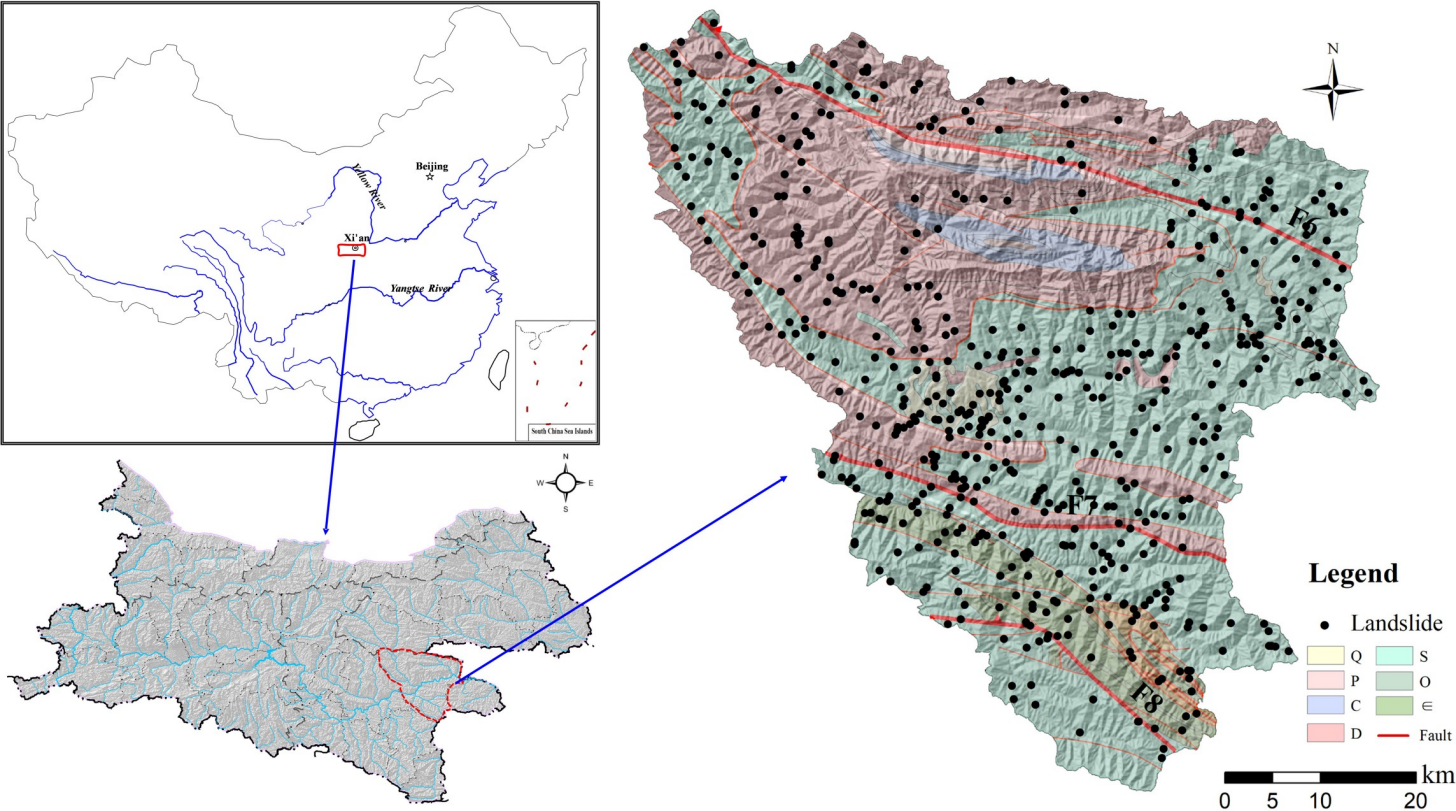
Location and geological characters of the study area.

The study area is located at the Xunyang District of the southern part of the Qinling Mountain Range, ranging from 108°58′~109°48′E and 32°29′~33°13′N. The altitude ranges from 185~2358.4 m (a.s.l.) in the area and the Han River flows across the Ankang basic between the northern Qinling Mts. and southern Daba Mts. in a NW-SE direction. The entire Han River catchment of the study area covers an area of 3550 km^2^, as presented in Fig 1. The geomorphology of the area can be characterized by steep slopes in the northern part and relatively lower slopes in the Daba Mountain Range in the south, encompassing the flat Han River basin. The climate is northern subtropical and has an average yearly rainfall amount of 730.9 mm, according to recorded rainfall data from 1973 to 2013. The rainfall is mainly concentrated in the June-September time frame each year, and the mean monthly rainfall amount can reach 137 to 146 mm, which induces hundreds of landslides along the catchment. The land use types on the mountain terrain are mostly forest.

The outcropped stratigraphy in the study area primarily include the Upper Carboniferous limestone of Yangshan Group (C3y), the Upper Devonian phyllite and limestone of Shijiagou Group (D3s) in the Qinling Mts of the northern part, and the Lower phyllite and mica schist of the Banjiuguan Group (S1) in the Daba Mts of the southern part. From the perspective of tectonic units, the region is located between the southern Qinling orogenic belt and the Daba arc-shaped faults, and is on the Xunyang-Ningshan nappe structure between two major faults in the area, the Zhen-an Baihe (Fig 1), mainly extended in a NW-SE direction and encompassing several secondary faults between them. These faults largely control the spatial extension of geomorphological features and flow directions. Holocene diluvium and residual sediments of silt clay with gravel on the slope can be prone to shallow landslides triggered by rainfall. It is commonly considered that multi-phase tectonic stress fields are complex and result in extremely fractured and weathered rock mass (e.g., particularly for formations consisting of Ordovician sericite phyllite and schist), which significantly increase the potential to landslides and rock fall hazards. Therefore, some research has emphasized the mechanism in tectonic controls on the spatial distribution of the landslides [18], and have pointed out that landslide distribution is strongly linked to spatial extension of the tectonic faults. Also, the spatial relationship between tectonic environment and landslide distribution is the main focus of the research.

**Fig 2:**
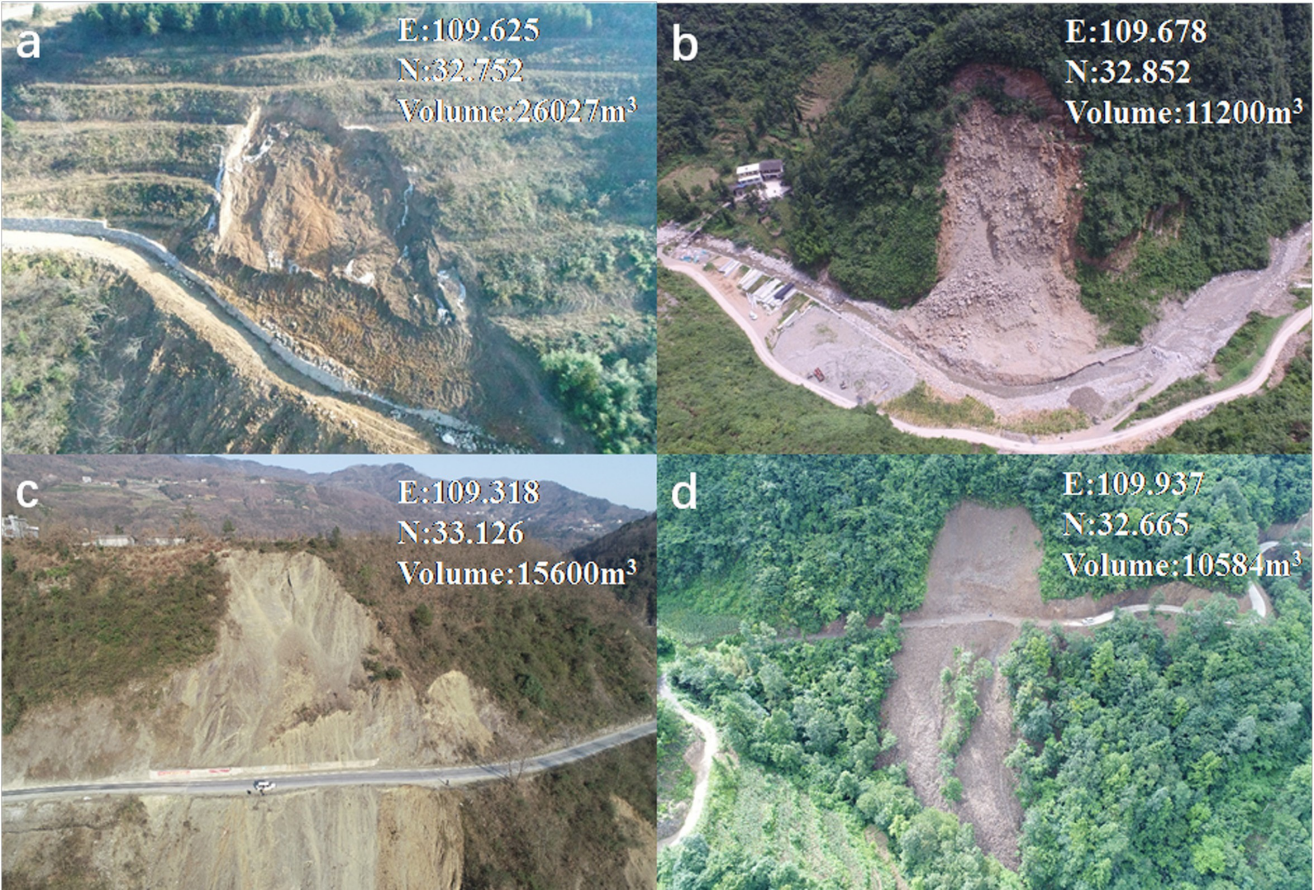
Typical landslides in the study area: (a) Yujiaya Landslide; (b) Lijiaping Landslide; (c) Douchuankou Landslide; (d) Zhongmiao Landslide. (The pictures was obtained by using the UAV aerial photography technique by ourselves.)

Data inventory of landslide distribution in the Xunyang District was constructed through field investigation and visual interpretation of the remote sensing images of the landslides at multi-phases. The discrimination of a landslide scar through remote sensing image interpretation was judged by the barren land in which vegetation vanished and by local subsidence of the ground (i.e., sunken topography). Although the volume of the landslide is not large (e.g., measured volume of most landslides are less than 2*104 m^3^), the threats generated by the extensively occurring landslides have caused risks to the public traffic and town safety due to abrupt and concentrated landslides during periods of extreme rainfall. The deposits of unconsolidated landslide material can evolve into debris flow under extreme rainfall along a steep gully. After field-based geological hazard investigations, 556 landslides were identified, and 541 landslides can be classified as shallow landslides as a result of rainfall infiltration into unconsolidated slope deposits. The remaining rock failures are associated with the stratigraphy composed of jointed and weathered phyllite and schist. The failure types of shallow deposits under rainfall are commonly observed as translational types, and the failure plane is generated along the rock-soil interface or the maximum reaching depth of the wetting front (Fig 2). Also, the rock slope failure was controlled by the structure plane, referring to the joints and cracks. From a geological point of view, landslides are more densely distributed within the southern part where sericite phyllite of the Banjiuguan Group (S1) dominates, and, therefore, the fractured rock mass controls its long-term slope stability.

## 3. Materials and methods

The WoE method was proposed based on information and Bayesian theory, by using a bivariate statistical method. It was previously applied to explore potential gold distribution and mapping [19]. Afterwards, the WoE method has been extensively applied to the fields of landslide susceptibility zonations, and the method has been considered as a reliable and cost-effective technique compared with the field observation and other deterministic methods [20–22]. The general workflow is composed of two major assessments: the probability of event distribution for a certain class of a factor, and the overall probability for the event distribution [20]. In statistical analysis for landslide susceptibility zonation, two main assumptions constitute the most crucial principles behind. In detail, predicted landslides in the future will occur under similar conditions compared with that of the landslides occurred in history, in which only a specific landslide type can be discussed in the analysis. Secondly, the causative factors for landslides susceptibility zonation remain constant during entire analysis period, which emphasizes its inherent geologically driven forces leading to slope instability in a long-term scale. Therefore, above-mentioned landslides distribution can be used to determine prior probability and conditional probability of the landslide events (i.e., posterior probability) in this Bayesian method. Prior probability is commonly determined according to inventory data of historical landslide distribution, named as P(E), and it can be influenced by an additional causative factor Fi. Thus, the posterior probability referring to the landslide contribution due to the presence of factor class Fi can be delineated as Eq (1):

$$P\left(E|F_{i}\right)\text{=}\frac{P(E)\cdot P(F_{i}|E)}{P\left(F_{i}\right)}$$ (1)

By overlapping the landslide inventory map with each causative factor map, spatial statistical relationships can be obtained and used for quantifying the effects of classes of the causative factors on the landslides by assigning positive or negative weight. Here, potential occurrences of landslides in the future will be considered under either presence or absence of a causative factor class Ei by using a pair of likelihood ratios, as shown in Eq (2):

$$W_{i}{}^{+}\text{=In}\frac{P\left\{E_{i}|I\right\}}{P\left\{E_{i}|\bar{I}\right\}}W_{i}{}^{-}\text{=In}\frac{P\left\{{\bar{E}}_{i}|I\right\}}{P\left\{{\bar{E}}_{i}|\bar{I}\right\}}$$ (2)

In Eq (2), P{E_i_│I} represents the conditional probability of landslide occurrence in the presence of factor class E_i_ (i.e. the landslide area in the domain occupied by the i-th class of factor E) under given landslide events I (i.e. total landslide area in the study area), and P{Ei│Ī} is accordingly about the conditional probability of the landslides under the presence of factor class E_i_ when landslide events I does not happen (i.e., Ī refers to the total stable region without landslide events). Parameter W_i_^+^ refers to the weight assigned to a certain raster referring to the influence of a factor class E_i_ on landslide events. Accordingly, W_i_^-^ is the weight assigned under the absencesss the factor class Ei, and the parameter Ē represents the remaining classes of the factor E. Then, for a specific raster unit receiving the impacts of certain factor classes, parameter C is introduced as the summation of W^+^ and W^-^ considering the effect of the i-th class of the causative factor E, and also the impacts of the absence of other sub-classes, which will be used for the identification of overall weights assigned to a raster unit, as described in Eq (3):

$$\text{C=}W_{i}{}^{+}+\left[\sum_{j=1}^{n}W_{j}{}^{-}\right]-W_{i}{}^{-}$$ (3)

In Eq (3), the parameter of $W_{\text{i}}{}^{+}$ has also the same meaning as they are indicated in Eq (2). The term of $\left[\sum_{j=1}^{n}W_{j}{}^{-}\right]$ represents the summation of the weights calculated for the absence of all the sub-classes for a specific raster unit. $W_{\text{i}}{}^{-}$ indicates the influence weight for the absence of factor i-th class.

As mentioned earlier, landslide occurrences can be predicted according to knowledge on the past landslides contributed from each controlling factor, also named as the evidence, thus, different groups of factors have distinct effects on landslide occurrences. The contribution of factor classes, also named as the information value obtained by using the WoE method, can be described as Eq (4), where I(Y, x_1_, x_2_, …, x_n_) represent the evidence provided by the groups of geological factors, and P(x_1_, x_2_, …, x_n_) is the probability of a landslide occurrence considering effects of groups of selected factors, and P(Y) is the overall landslide probability.

$$I\left(\text{Y,x}1,\text{x}2\ldots \ldots \text{xn}\right)\text{=}\ln\frac{P\left(\text{x}1,\text{x}2,\text{x}3\ldots \ldots \text{xn}\right)}{P\left(Y\right)}$$ (4)

Here x1, x2, …, xn represents different predictive variables, Y indicates the landslide events.

In WoE method based on Bayesian principle, an optimal set of landslide causative factors should be appropriately selected to reflect its indispensable effects in determining the spatial distribution of the landslides in a region. As mentioned above, an assumption of conditional independence of factors should be predetermined before performing landslide susceptibility analysis. Some researchers have indicated that an increasing number of predictive variables raise the possibility of conditional dependence and result in unreliable outcomes [23]. For example, geomorphological attributes of the curvature, was strongly related with local slope angle and relative position of the slope. Although a landslide generation process is commonly considered to be a complicated phenomenon, and some factors are correlated when handling specific landslide triggering process. To ensure conditional independence of each factor and avoid deriving a problematic regional landslide susceptibility map, it is worth noting that a correlation test is crucial to remove correlative factors, prior to overlapping each weighted factor map. If the correlation index between each group of two factors is higher than the threshold, the factor with more predictive power on the landslides occurrence can be retained and adopted in the model. Therefore, a widely-used method for the assessment of conditional independency for pairs of causative factors in this research is chi-square statistic test, which is related to the variation between expected and observed frequency in landslides occurrence with respect to a certain factor. Detailed description about the testing method can be seen in some previous published literature [23, 24].

The DEM data, freely downloaded from the data source of SRTMDEM (USGS, resolution: 30m) of the entire area was used in the susceptibility analysis, and it was furthered processed for deriving geomorphological attributes, including slope angle and slope curvature and even flow network by applying the basin hydrology spatial analysis tool in ArcGIS software. The stratigraphy and tectonic lineament were reconstructed by digitalizing the geological map of Xunyang District at a scale of 1:200000 obtained from the Shaanxi Geology survey. Precision of rasterized data of stratigraphy map was in accordance with that of the DEM data mentioned. For the lineament of tectonic faults, the parameter of the distance to faults was adopted in the study to understand the effect of fault on the distribution of the landslides. Another lineament factor of road, the density of road was considered for quantifying the effects of engineering activity on the landslides.

Total 556 landslides were investigated through both field identification and remote sensing interpretation work (Landsat 4-Band 2, resolution: 60 m, provided by Geospatial Data Cloud site, Computer Network Information Center, CAS) and recorded for the inventory map. Due to relatively small scale of the landslides volume (i.e. generally smaller than 1*10^4^ m^3^), identification of the shallow landslides through the remote sensing images was performed by comparing the barrenland of landslide scars and its surrounding vegetation area. Most of the landslides investigated in this area was mostly triggered by long-lasting heavy rainfall events and engineering activities. Based on the results of field investigation in the study area, typical failure type of the landslides can be classified as shallow translational sliding within deposits or along the interface between upper deposits and lower weathered layer. Then, 70% of the total number of landslides were classified into group for establishing the relationship between spatial landslides and each factor class, the remaining dataset of landslides was adopted for testing the weights determined for each factor by using the ROC curve. The separation of the dataset was randomly performed.

**Fig 3:**
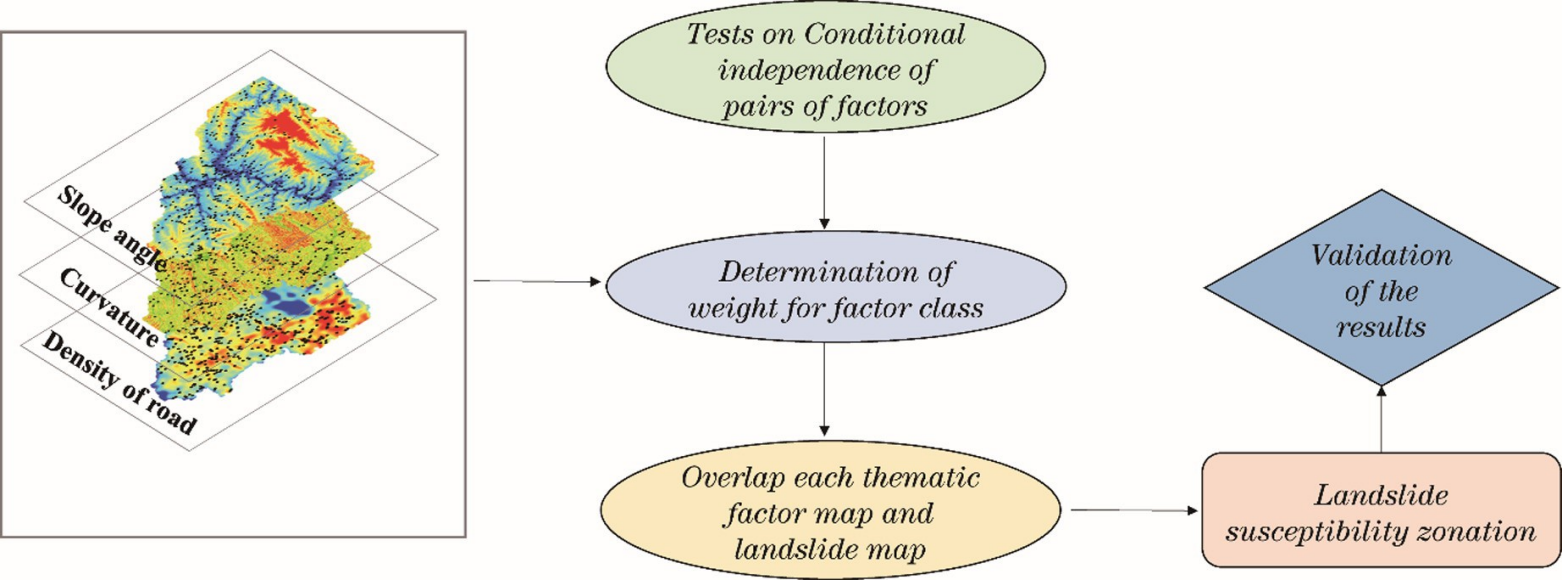
Flow chart of the Weight of Evidence method for a landslide susceptibility analysis.

The general working flow of the research can be described as shown in Fig 3.

## 4. Thematic map of controlling factors

Before combining each weighted factor map for performing the LSA analysis, conditional independence test was conducted for each pair of causative factors. If correlation coefficients calculated for pairs of factors exceeding the thresholds aforementioned, the total number of causative factors will be diminished according to the assumption of the WoE method. Results presented in Table 1, have revealed that the factors of slope angle and curvature have a very strong spatial correlation, as shown by the correlated factor of 0.67. In contrast, the factor of stratigraphic unit have scarcely spatial correspondence with other factors, because the spatial distribution characteristics of exposed stratigraphic successions have no inherently derivative relationship with other factors. On the other hand, a coincidence happens between the density of road and the density of flow network. The correspondence between these two factors was based on the fact that main road networks constructed in the area commonly extend along the paths of the rivers along which towns and villages of moderated-large sizes are located. Then, in this research we considered only the factors of slope angle, slope aspect, stratigraphic units, distance to faults and the density of road network. The reason why we remove the influence of the density of flow network, attributing to limited variation in water level of the river and thus providing ignorable effect on the slope stability. However, the road construction process significantly alter the structure and, thus the strength of the slope material. Therefore, factor of the density of road network was considered as a crucial factor in this research.

**Table 1:** Correlation coefficient of different pairs of landslide causative factors.

	Slope angle	Slope aspect	Slope curvature	Stratigraphic unit	Distance to faults	Density of river network	Density of road network
**Slope angle**	1						
**Slope aspect**	0.4	1					
**Slope curvature**	0.67	0.33	1				
**Stratigraphic units**	0.29	0.22	0.36	1			
**Distance to faults**	0.25	0.22	0.31	0.52	1		
**Density of river network**	0.37	0.29	0.41	0.35	0.49	1	
**Density of road network**	0.46	0.21	0.44	0.49	0.31	0.69	1

The maps for each landslide controlling factor were reconstructed and overlapped with the landslide distribution in determining the weight of each contribution (e.g. relative importance degree) to the initiation of the landslides. In general, three types of datasets should be adopted and applied in the analysis: (a) the first was based on elevation data represented by using the raster dataset of the study area, and it can be adopted for deriving the slope angle, slope aspect, after applying a spatial hydro-geological analysis; (b) the second category refers to geological conditions including the stratigraphic succession and tectonic faults, (c) the third category of triggering factors mainly includes the road network, which represents the impact from engineering activity. It is worth noting that the discrete data (e.g., stratigraphic map) have different data interval-division methods compared to the continuous data.

### 4.1 Geomorphological factors

**Fig 4:**
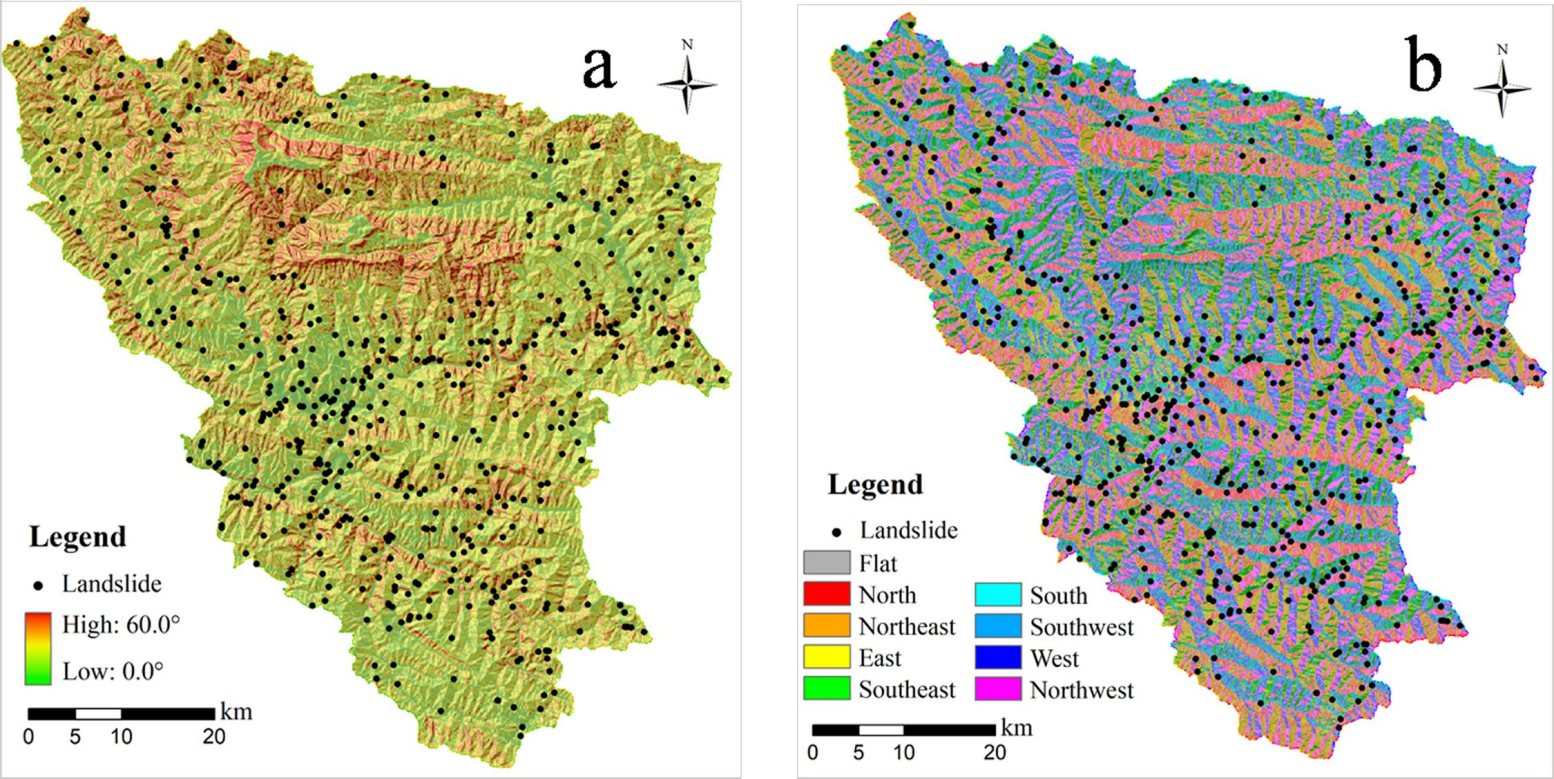
Spatial relationship between controlling factors and landslide distribution: (a) slope angle; (b) slope aspect.

The spatial relationship between each factor map of the slope angle and the slope aspect, and landslides reflecting the effects of geomorphological features in the study area are shown in Fig 4A and 4B, respectively. All these thematic maps can be directly derived from the DEM data by simple data processing methods. Slope angle has impacts on underground flow and internal stress in slopes, meanwhile, the deposition of the slope soil requires an appropriate condition of slope angle range, and an inclined slope provide a gravity-driven force to initiate a landslide process. Therefore, landslides occur more frequently on slopes with slope angles of 20 to 40°. The fact that landslides typically do not occur near the top of slopes is related to loose soil layers not being to accumulate on slopes with higher slope gradients. However, it is worth noting that a higher correlation value was seen between the factor of elevation with slope angle and slope aspect after chi-square tests and was able to be ignored. Correspondingly, the shape of a slope can affect the rainfall infiltration path and stress distribution, particularly inducing a stress concentration effect, thus the stability of a landslide.

In addition, some considered causative factors do not straightforward induce the initiation of mass movement, for example, the slope aspect can be derived from DEM data and reveals solar radiation and humidity conditions, which highly influence the vegetation growth on the slope surface and consequent slope stability. The slope facing towards the south direction generally receives much more energy than those on the north-facing slope, which facilitates the growth of dense vegetation. However, an intense weathering process can also be highly experienced by the rock mass on the south-facing slope, resulting in a fractured slope structure and increasing landslide probability.

### 4.2 Geological factors

**Fig 5:**
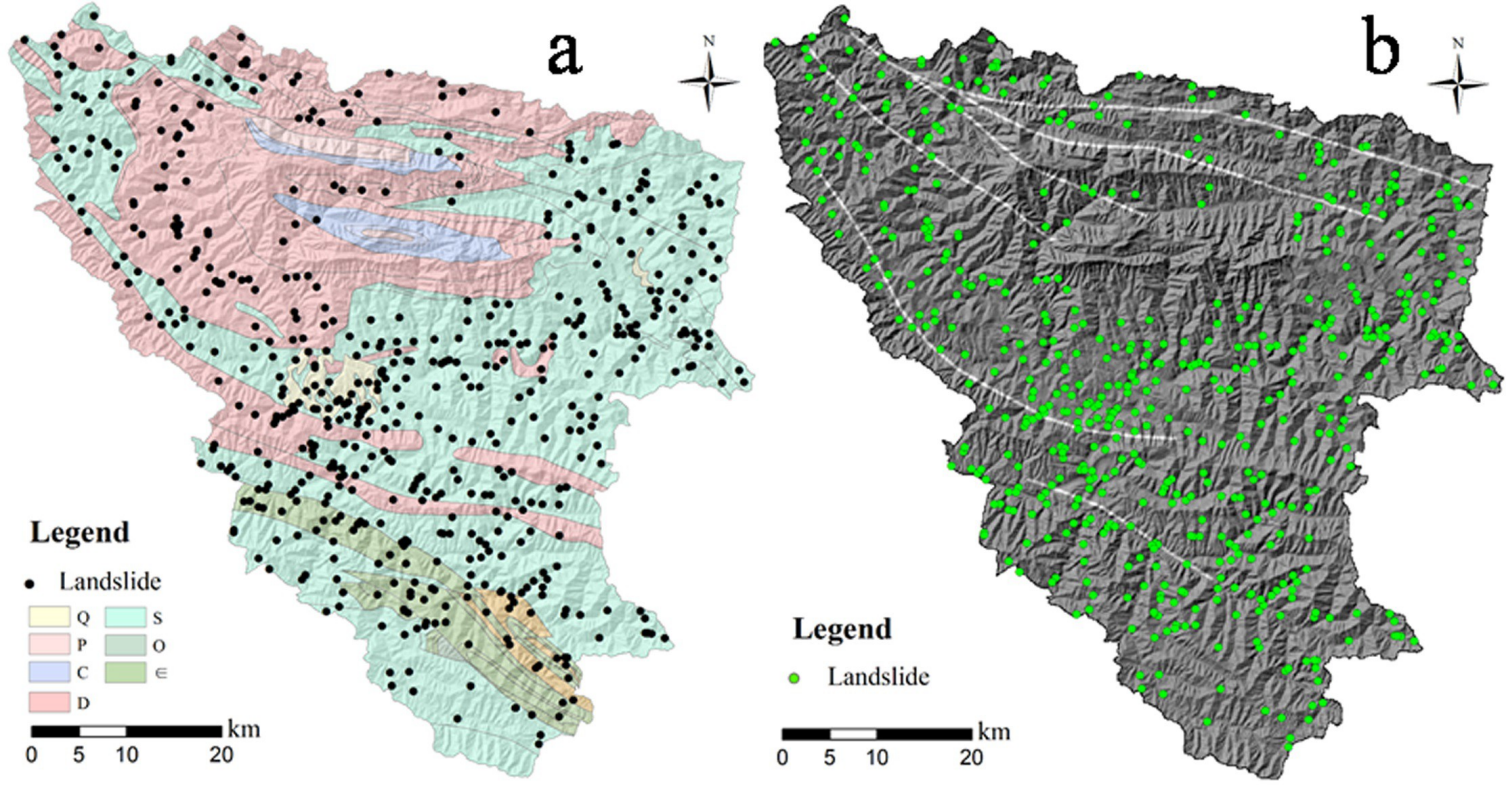
Spatial relationship between geologial factors and landslide distribution: (a) Spatial relationship between stratigraphy and landslides; (b) Spatial relationship between lineament of tectonic faults and landslides.

Geological conditions have been considered as the most significant factors inherently driving the landslide occurrence. A specific combination of lithological types presents unique strength and slope structures, which largely determine the deformation characteristics of soil-rock mass and the movement process of the slope failure. Due to contributions from multi-phase tectonic deformations (e.g, wrinkles and faults), which will be discussed in the following text, the rock mass was fractured and presented degraded failure strength after a long-term weathering and leakage process. Particularly in this area, as shown in Fig 5A, investigated landslides generally occurred on silurian strata mainly exposed in the southern area, consisting of the Banjiuguan group of lamellar sericite phyllite and carbonaceous slate and the Meiziya group of schist. From a regional point of view, the spatial distribution of landslides in a catchment has a strong relationship with the extension of highly fractured stratum with low strength, and approximately 73% of all landslides were generated on those stratigraphy. Otherwise, landslide seldomly occurred on thick layer strata of carbonatite of the Carboniferous and Devonian systems, which present a relative dense texture and weak moderate weathered features.

The lineament of tectonic faults is considered as also a significant factor in controlling the landslides and commonly resulted in fractured strata and accelerated the weathering process. Field investigation results reveal that spatial distribution of the landslides highly extend along major tectonic faults, particularly referring to the Ankang-Shenhezhen anticlinorium and Gongguan-Baihe fault at the southern part of the study area. Apparently, the spatial distribution of landslides is more intensive when having an overall shorter distance to the fault structure (Fig 5B). However, in northern areas in which intact sedimentary strata predominate, there is no significant tectonic faults traversing across the area and landslides seldomly occur. Therefore, in this study, the distance to fault was adopted as a quantitative factor of the influence of faults on landslide distribution. Buffer zones with various distances to the fault were set in ArcGIS 10.2 software to calculate the relative importance of the sub-classes related to this factor.

### 4.3 External triggering factors

**Fig 6:**
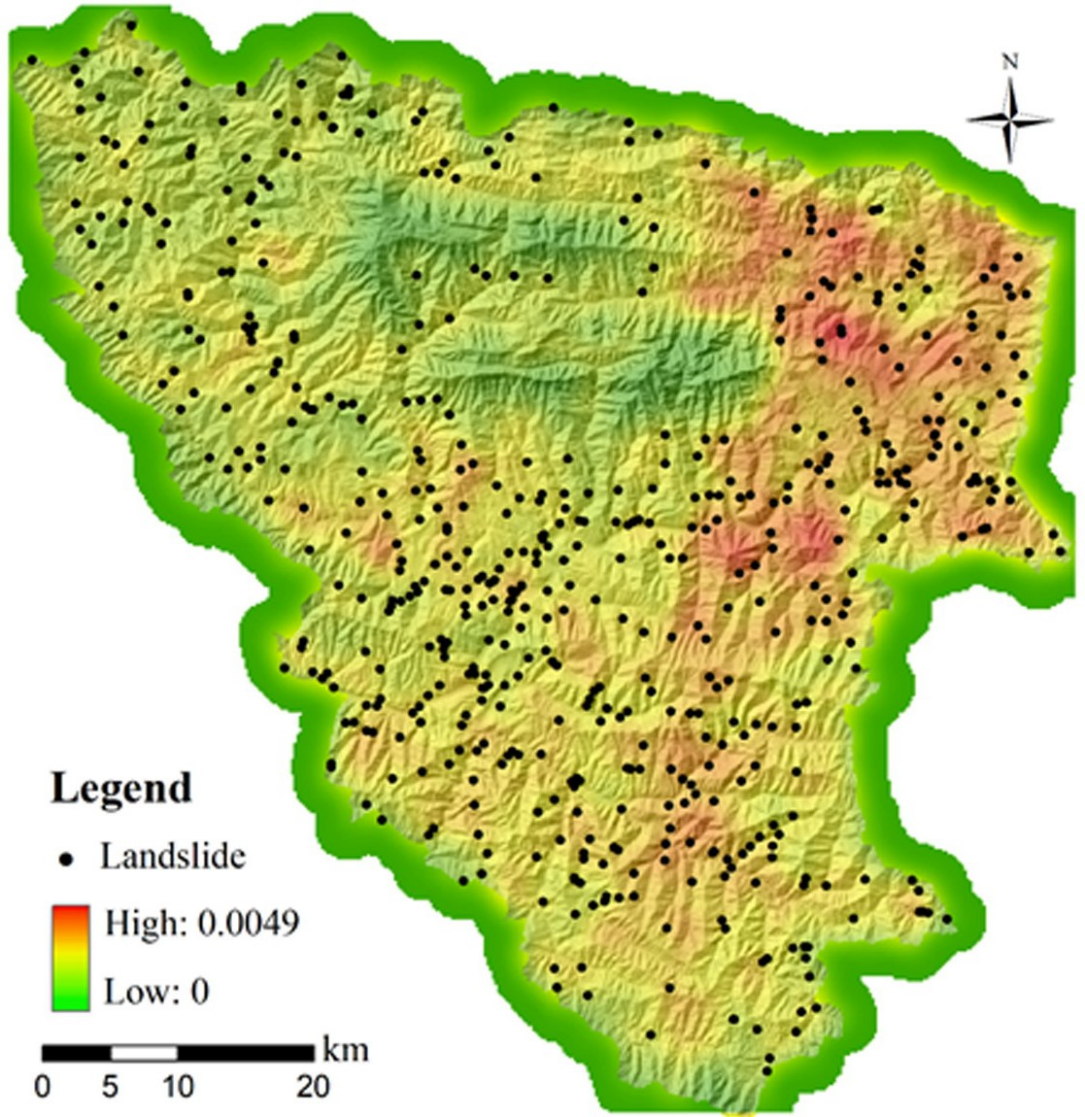
Spatial relationship between road density and landslides.

The above-mentioned factor can be attributed to internal factors predetermined by the geological conditions in a long-term scale. External engineering activities and rainfall distribution were also considered as critical triggering factors for landslides. The anthropogenic engineering constructed in mountainous areas have largely altered original geomorphological features and imposed additional engineering loads on the slope, which highly increase the probability of a slope being prone to instability. To be specific, frequent construction of expressways and railways in recent years in the Xunyang District have accompanied the slope cutting campaign and vibrating loads from the vehicles on the road, which have considerably weakened the strength of the slope material and altered initial stress field of the slope. Consequently, this has led to a cluster of landslides in the area. In this regard, the density of road network was adopted as a factor in this research, which represented the intensity of engineering activity. The increased frequency of landslides are highly related to the intensive engineering activities(Fig 6).

## 5. Results and discussion

By applying the WoE method, weight value of each sub-class of a factor can be determined and the results illustrate their relative importance in causing regional landslides. Table 2 lists each parameter used or calculated landslide proportion in each causative factor class, which can be further applied by using the WoE method to determine the value of each weight. The obtained weight value clearly reveals the spatial relationship between landslide distribution and each landslide factor class. In detail, almost each sub-class of stratigraphic groups and distance to faults have positive influence on the landslide occurrence in accordance with calculated weight values introduced as mentioned in previous section. However, positive values obtained in each sub-class probably demonstrate that the landslide has been randomly distributed in different categories of a single factor, without a concentration in a specific range. Thus, a more powerful indicator, referring to parameter C as mentioned previously, (i.e., if parameters of w^+^/w^-^ > 0, then c>0; parameters of w^+^/w^-^ < 0, then c<0), a higher value assigned to a grid indicates a stronger relationship between causative factors to a landslide and a higher potential to future landslides. In contrast, causal factors including slope angle, curvature, density of river network, and density of road networks present variable weight values of factor classes, some of which are highly related to the occurrence of landslides and some present little correlation with landslides. For example, landslides commonly occurred at slopes with slope angles ranging from 20 to 40°, and at northern and northeastern-facing slopes, consistent to what has been described in the relationship in section 4.

**Table 2:** Weights of each landslide controlling factor and its sub-classes by the WoE method.

Types of landslide control factors		Sub-division classes	Number of pixels of landslides within sub-class	Number of pixels of each sub-class	Proportion of landslide area in a factor class
Geomorphological factors	Slope angle	<10°	7	87783	-2.121
		10~20°	45	55220	-0.815
		20~30°	210	23483	0.144
		30~40°	230	413577	0.567
		40~50°	60	130717	-0.615
		50~60°	32	11293	-1.205
		>60°	23	291	-1.870
	Slope aspect	45~135°	152	89340	1.177
		135~225°	187	90704	1.869
		225~315°	148	87395	-0.752
		315~45°	121	86902	-0.872
Geological factors	Stratigraphic Unit	Hard Carbonatite rock	27	13190	0.173
		Moderate weathered rock	179	175605	0.020
		Metamorphic rock	402	165499	0.817
	Distance to faults	<200 m	356	7505	0.172
		>200 m	252	346857	0.025
Anthropogenic activities	Density of road networks	0~0.0005 km/km^2^	15950	12	-1.632
		0.0005~0.001 km/km^2^	32654	88	-0.764
		0.001~0.0015 km/km^2^	29995	197	1.285
		0.0015~0.108 km/km^2^	1538	311	2.433

However, it is worth noting that some research has pointed out a correlation of a certain factor class with landslide distribution is not necessarily linked to the initiation of a landslide, and it may coincide with classes of other causative factors, which are possibly the major causes for the intrinsic driven force of a landslide [23, 24]. For example, in regions where weak strata of sericite phyllite and schist belonging to epimetamorphic rock predominate, the landslides are concentrated under conditions of relative lower slope angles. In contrast, the northern area in which carbonatite strata are widely exposed is characterized by steeper and higher slopes (Fig 4b), but the area was inversely impacted by mass movement. In other words, it is commonly appropriate to consider effects of multiple factors contributing to the occurrence of landslides, but not relying on a single factor in an ordinal scale. Moreover, sub-classes of a factor may involve a relatively small area and coincidentally cover an area in which landslides occur, which possibly leads to an extremely high value of positive weight. However, it does not representatively refer to the impact of the factor, and it can lead to unreliable outcomes.

**Fig 7:**
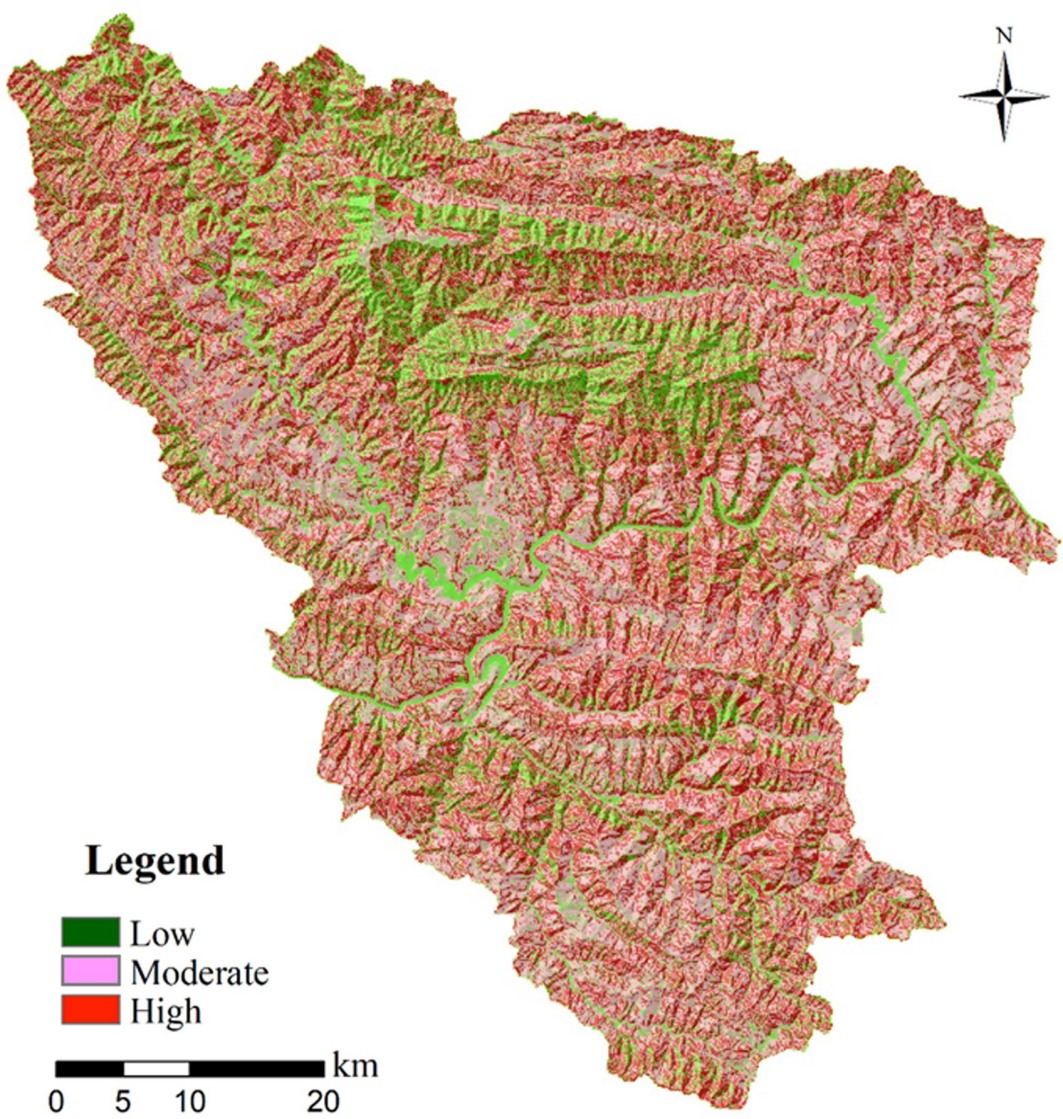
Landslide susceptibility map derived using the WoE method.

Afterwards, modelling outcomes of a landslide susceptibility map can be obtained through superposition of each weighted factor map, and the total information value can be derived by integrating each of information value of all the factor class given in a certain area (See Eq(3)). The summation of the weights, also called the Landslide Susceptibility Index (LSI), was analyzed to understand the probability of landslide occurrence in a given area. According to results of the summation of each weighted factor map carried out by using the raster calculation function in ArcGIS software, total information values ranging from -13.897 to 13.456 were obtained over the entire study area. According to statistical results (Table 3) obtained by applying the spatial analysis function in the ArcGIS software, a “High” landslide susceptibility zone occupies 49.57% of the total area, covering 60.17% of the total landslide quantity. In contrast, a “Low” susceptibility zone covers 8.16% of the total area and only includes 4.33% of the total number of landslides. The results reveal that the model performed considerably well in predicting the landslide probability since most of the landslides from the training dataset fall into the category of the high susceptibility area. To be more specific, high susceptibility zones (Red color range shown in Fig 7) are more concentrated within epimetamorphic stratigraphic successions, mainly weak Ordovician sericite phyllite of along the lineament of several main river flows of the studied area.

**Table 3:** Statistical results for each landslide susceptibility zone.

Evaluation method	Susceptibility zone	Total area (km2)	Proportion of each zone to total area (a)	Quantity of landslides in each zone	Proportion of landslides to total landslides (b)	b/a
Weight of Evidence (WoE)	High	1735.52	49.57%	395	0.6017	1.21
	Moderate	1369.37	42.30%	137	0.3550	0.84
	Low	369.35	8.16%	34	0.0433	0.53

**Fig 8:**
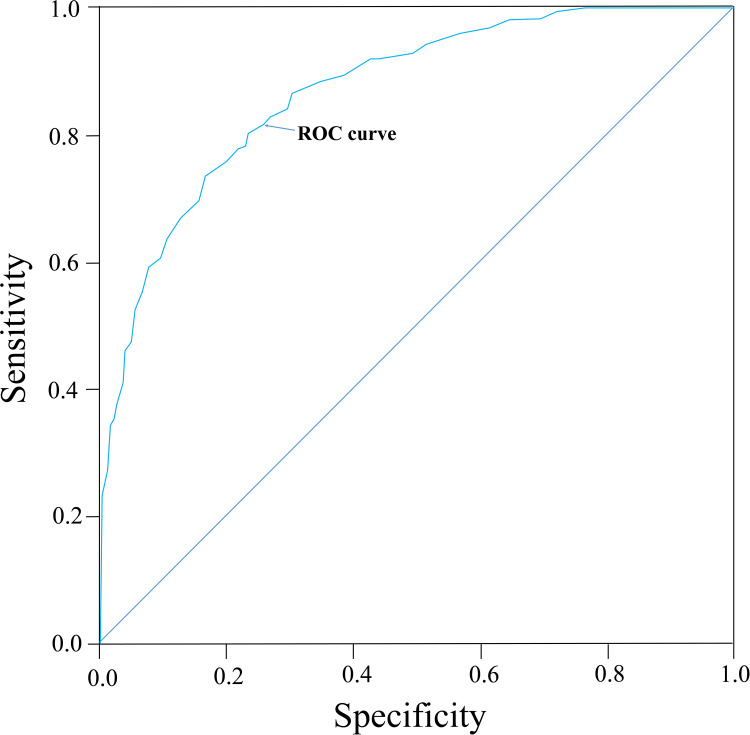
Validation of WoE method using the ROC curve.

However, a classification of the landslide susceptibility zonation merely based on discrete values of the LSI index is commonly subjective, and it is difficult to find an explicit boundary and clear relationship among these values for determining different levels of landslide susceptibility zonation. In many previously published works, the Receiver Operating Characteristic (ROC) curve has been used as an efficient method for evaluating or validating the predictive power of statistical models by using testing landslide inventory, which was not involved in constructing the prediction model. The Y-axis of the curve is True positive rate (i.e., success rate), indicating “correct prediction” of the accumulated percentage of landslide affected area derived from the model in comparison with the total landslide area in a certain factor class range, and the X-axis of the curve represents the false positive rate (1-specificity), which indicates the ratio of the cumulative non-landslide area to the total non-landslide area within a given range of factor class. The separated set of landslide dataset, which was not used in the modelling process of the WoE, was tested in the validation stage compared with the predictive capability of the WoE model. The training set was sampled randomly due to uncertainty over the accurate recording time of landslide occurrences. The principle of validation method for a susceptibility map is to introduce the concept of prediction rate [8], which delineates which percentage of validating set of landslides falls into the category of the high susceptibility zone. Thus, the validation set of the landslides overlapped with the obtained susceptibility map, for the aim of obtaining a ROC curve consisting of the cumulative landslide area in each susceptibility level, and corresponding total area, as shown in Fig 8. Results indicate that 91.87% of the cumulative landslide area falls into the high and moderate susceptibility zone in the study area, and it provides a relatively good fit of the model.

The landslide susceptibility map provided using the WoE method for the Xunyang District, Shaanxi Province were processed and obtained, involving a series of causative factors which were inherently related with geomorphological and geological characteristics of the study area. It was evident that external triggering factors, such as anthropogenic activities, highly affected the stability of the slope through changing internal stress fields and strengths of the slope materials. However, the mechanism behind a landslide varies upon different slope conditions due to distinct geological conditions. From a regional point of view, geomorphological factors (slope angle, and slope aspect) and geological factors (stratigraphic categories) significantly control the spatial distribution of landslides as inherent driven forces. Compared with the influencing power of external triggering factors, regional landslides were distributed in accordance with the range in critical slope angle and extension of major faults in this research, which emphasizes that geological conditions significantly control the high probability occurrence of the landslides. In addition, more landslides occur in weak and fractured epimetamorphic strata. A landslide susceptibility map was suggested as a statistical method referring to the delineation of different levels of spatial probability for landslide occurrence. It should be noted that the selection of causal factors of landslides was not invariant and depends on the model selected and the range of each factor map, which both influence the results of landslide prediction.

## 6. Conclusions

In this research, landslide susceptibility assessment using the Weight of Evidence (WoE) method for the Xunyang District of the Qin-Ba Mountain Region was successfully conducted, which could be further processed for regional landslide risk evaluation. Multiple landslide causative factors were collected and compared for the aim of quantifying each independent contribution to the landslide initiation. Compared with the method of frequency ratio, the predictive power of the WoE method can be further identified for each causative factor, through determining the correlation between each factor class and past occurred landslides. A certain range of factors, including the slope angle, stratigraphy and lineament of anthropogenic activities have presented high probabilities of landslide-prone areas, and, thus, geomorphological factors, lithology, and external triggering factors, including anthropogenic activities significantly determine regional landslide distribution in the study area. However, the selection of landslide samples, resolution of DEM data, and the scale of the study area are also crucial reasons for the derived outcomes of the susceptibility map.

## Supporting information

S1 Data We have uploaded original DEM data and the landslide location (shape files) available in the ArcGIS environment as a supporting information file. (RAR)
